# Synergistic influence of cytokine gene polymorphisms over the risk of dementia: A multifactor dimensionality reduction analysis

**DOI:** 10.3389/fnagi.2022.952173

**Published:** 2022-10-26

**Authors:** Teresa Juárez-Cedillo, Nancy Martínez-Rodríguez, Gilberto Vargas-Alarcon, Enrique Juárez-Cedillo, Antonio Valle-Medina, Osvaldo Garrido-Acosta, Alfredo Ramirez

**Affiliations:** ^1^Unidad de Investigación Epidemiológica y en Servicios de Salud, Área Envejecimiento, Centro Médico Nacional Siglo XXI, Instituto Mexicano del Seguro Social, Mexico City, Mexico; ^2^Epidemiology, Endocrinology, and Nutrition Research Unit, Hospital Infantil de México Federico Gomez, Ministry of Health (SSA), Mexico City, Mexico; ^3^Department of Molecular Biology, Instituto Nacional de Cardiología Ignacio Chávez, Mexico City, Mexico; ^4^Sección de Estudios de Posgrado e Investigación, Escuela Superior de Medicina, Instituto Politécnico Nacional, Mexico City, Mexico; ^5^Facultad de Estudios Superiores Zaragoza, Universidad Nacional Autónoma de México, Mexico City, Mexico; ^6^Division of Neurogenetics and Molecular Psychiatry, Department of Psychiatry and Psychotherapy, University of Cologne, Köln, Germany

**Keywords:** polymorphism, cytokine genes, dementia, MDR, gene–gene interaction

## Abstract

**Objective:**

Evidence supports the important role of neuroinflammation in some types of dementia. This study aimed to evaluate the effect of epistasis of gene cytokines such as interleukin (IL)-α, IL-6, tumor necrosis factor (TNFα), and interferon-gamma (IFN-γ) on the susceptibility to the development of dementia.

**Materials and methods:**

In the study, 221 patients diagnosed with dementia and 710 controls were included. The multifactor-dimensionality reduction (MDR) analysis was performed to identify the epistasis between SNP located in genes of IL-α (rs1800587), IL-6 (rs1800796), TNFα (rs361525 and rs1800629), and IFNγ (rs2069705). The best risk prediction model was identified based on precision and cross-validation consistency.

**Results:**

Multifactor-dimensionality reduction analysis detected a significant model with the genes TNFα, IFNγ, IL1α, and IL6 (prediction success: 72%, *p* < 0.0001). When risk factors were analyzed with these polymorphisms, the model achieved a similar prediction for dementia as the genes-only model.

**Conclusion:**

These data indicate that gene–gene interactions form significant models to identify populations susceptible to dementia.

## Introduction

Dementia is a neurodegenerative disorder that results in a decreased quality of life and increased disability and mortality. The reported prevalence of dementia worldwide is varied; in Mexico, it ranges between 6.1 and 7.9% (Gutierrez-Robledo and Arrieta-Cruz, 2015).

The etiology of dementia is unknown; however, it has been related to both environmental and genetic factors (Harman, 2006; Fleming et al., 2022). Some cases are neuropathologically characterized by the presence of extracellular amyloid plaques and neurofibrillary tangles containing phosphorylated tau protein, followed by alterations and neuronal degeneration (da Cruz e Silva et al., 2010; Henriques et al., 2015).

The ApoE ε4 allele is currently the only genetic factor directly associated with the risk of dementia. Still, only 50% of the patients with dementia carry the allele, so other genetic factors must be related to this disease (Bertram, 2009; Zekanowski and Wojda, 2009; Nazarian et al., 2019).

Recent studies have reported that inflammatory responses play a key role in the neurodegenerative cascades of dementias (Heneka et al., 2015; Darweesh et al., 2018), and some of these markers have been associated with the severity and progression of the disease (Hesse et al., 2016; Olsson et al., 2016).

Genome-wide association studies (GWAS) support the interaction between the inflammatory process and dementia. In these studies, several inflammation-related genes have been identified. Among them are CR1 (complement receptor 1); MS4A (membrane-spanning 4-domains subfamily A), which crosses the membrane; TREM2 (triggering receptor expressed on myeloid cells 2); and CD33, mainly found in Caucasian and Chinese populations (Karch and Goate, 2015; Toral-Rios et al., 2015; Gillespie et al., 2022).

Other reported findings indicate that cytokines are key components of neuroinflammation and their levels are altered in patients with Alzheimer’s disease (AD). In addition, several case-control studies have been conducted to explore the relationship between cytokine polymorphisms and the risk of developing dementia.

Inflammatory markers, such as the tumor necrosis factor-α (TNF-α), interleukins (IL-1 and IL-6), and interferon-gamma (IFN-γ) have been reported as major molecules in inflammation. They are known to affect the brains of patients with dementia (O’Bryant et al., 2016; Zhu et al., 2017; Zheng et al., 2022). Cytokines, a large family of proteins, include interleukins, TNF, and IFN-γ. Given the cytokine response to inflammation, they have been classified as pro-inflammatory, such as IL-1, IL-6, and TNF-α, while others, such as IFN-γ, present anti-inflammatory properties (Zheng et al., 2016, 2022).

Then, some cytokine genes involved in inflammatory responses are linked to the susceptibility to dementia. They are highly polymorphic, and these polymorphisms affect cytokine expression (Bertram, 2009).

Since IL-1 has been linked to AD, any changes in the genes altering IL-1 production may be risk factors for the development of this disease (Rothwell and Luheshi, 2000). The IL-1 gene contains several polymorphisms; however, studies have only considered −889C/T to investigate the association with susceptibility to the disease.

The anti-inflammatory cytokine IL-6 is increased in dementia brains (Rubio-Perez and Morillas-Ruiz, 2012); nevertheless, some studies have demonstrated differential effects. Specifically, IL-6 has been shown to induce the cortical expression of the amyloid precursor protein (APP), while the overexpression of this cytokine suppresses amyloid β (Aβ) deposition *in vivo* (Chakrabarty et al., 2010).

TNF-α mediates the proliferation of neurons and induces the production of neurotoxic Aβ. Some polymorphisms of probable functional significance have been identified in the promoter region of the TNF gene, but the published results show conflicting data (Shawkatova et al., 2010; Ardebili et al., 2011).

IFN-γ may inhibit the secretion of soluble APP (sAPP). The IFN-γ gene is on chromosome 12 (Scott et al., 2000), with intronic-located polymorphisms. Some of these polymorphisms may affect IFN-γ secretion and the inflammatory process, triggering a pathologic change in dementia (Oda et al., 2002). Previous analyses have assessed the associations between polymorphisms of cytokine genes but individually.

We investigate the combinatorial effect of four (IL-1α, IL-6, TNFα, and IFN-γ) genetic polymorphisms involved in the inflammatory processes of microglial cells in the brain on the susceptibility to dementia in Mexican individuals.

## Materials and methods

### Study design, base, and participants

The population of this study comes from the Study of Aging and Dementia in Mexico (SADEM) (Juarez-Cedillo et al., 2012). A total of 931 individuals were included: 221 were diagnosed with dementia and 710 were controls (negative diagnosis for dementia or cognitive problems). Informed consent was obtained from all subjects involved in the study.

Dementia was diagnosed in two steps; the Mini-Mental State Examination (MMSE), along with a battery of neuropsychological tests, was first applied to identify older adults with cognitive problems. Second, dementia was confirmed by the consensus of a panel of experts. They followed the criteria for dementia in the Diagnostic and Statistical Manual of Mental Disorders (DSM-IV-TR) and the AD criteria proposed by the National Institute of Neurological and Communicative Disorders and Stroke and the Alzheimer’s Disease and Related Disorders Association (NINCDS-ADRDA) (McKhann et al., 1984). The Clinical Dementia Rating (CDR) (Hughes et al., 1982) was also used.

All the patients were born in Mexico; they became participants once they were informed of the study and provided their signed consent. The study was conducted according to the guidelines of the Declaration of Helsinki and approved by the National Commission of Scientific Research as well as by the IMSS Ethics Commission, registration number R-2015-785-012.

### Clinical characteristics and biochemical analysis

The interview specifically designed for this study included the following: age, sex, and body mass index (BMI) calculated as weight (kg)/height (m^2^) and divided into three groups, namely, normal weight (BMI < 25 kg/m^2^), overweight group (BMI of 25–29.9 kg/m^2^), and obesity (BMI > 30 kg/m^2^).

Venous blood samples from the subjects were obtained after fasting (10 h). Glucose, triglycerides, total cholesterol, and high-density lipoprotein cholesterol (HDL-c) were determined using an Ekem Control Lab semi-auto chemistry analyzer. The Friedewald equation and the De Long modification were used to calculate the low-density lipoprotein cholesterol (LDL-c) (Romano et al., 1993).

### Genetic polymorphism detection

Genomic DNA from peripheral blood was obtained using the Lahiri and Numberger method (Lahiri and Nurnberger, 1991). The DNA purity was verified in a NanoDrop^®^ 1000 spectrophotometer at a wavelength of 260 and 280 nm. To identify potential gene-gene interactions, the SNPs were selected considering prior publications and those previously reported on the SNP website.^1^ They showed an LD threshold of r2 ≤ 0.1 and a minor allele frequency (MAF) ≥ 1%; they were also clinically relevant to neurological diseases. Quality control procedures included MAF (Skol et al., 2006) and the Hardy-Weinberg equilibrium (HWE) (*P* < 0.05) (Ryckman and Williams, 2008).

The SNPs IL1-α rs1800587, IL-6, rs1800796, TNF-α rs361525, rs1800629, and IFN-γ rs2069705 were selected from the four cytokine genes included in this study (see Table 1).^2^ Their frequencies for Africa, Asia, Eastern Europe, South Asia, and the United States have been reported by the 1000 Genome Project (Genomes Project et al., 2012). In addition, the biological effects of these variants and their link with AD were considered.

**TABLE 1:** Reference SNP (rs) report.

Gene	dbSNP^a^	Allele	Genomic location	Location in gene
IL1-α	rs1800587	G > A	2:112785383	Upstream transcript variant
IL6	rs1800796	G > C	7:22726627	Non-coding transcript variant, intron variant
TNF-α	rs361525	G > A	6:31575324	Upstream transcript variant
	rs1800629	G > A	6:31575254	Upstream transcript variant
IFNγ	rs2069705	G > A	12:68161231	Upstream transcript variant

^a^SNP ID in database db; SNP. Given name according to NCBI.

IL, interleukin; TNF-a, tumor necrosis factor-alpha; IFNG, interferon-gamma; TGFB1, transforming growth factor beta 1.

The samples were genotyped using a real-time PCR system according to the manufacturer’s instructions (Applied Biosystems, CA, USA). Standard DNA controls were used in each real-time PCR assay.

### Statistical analysis

The statistical analysis was performed using IBM SPSS Statistics version 23.0 (IBM Corp., Armonk, NY, USA). The Student’s *t*-test was used for continuous variables, and the results were expressed as the mean standard deviation, while categorical variables were presented as *n* (%). Fisher’s exact test was used to compare allele and genotype frequencies between cases and controls. Hardy–Weinberg equilibrium was tested by Fisher’s exact test *X*^2^ analysis for a 2 × 2 table. The odds ratio (OR) was calculated to evaluate the correlation between the genotypic/allelic frequencies of each gene and the susceptibility to develop dementia. It was adjusted for sex, age, and other factors that showed a statistically significant association with susceptibility to dementia. Bonferroni test was applied. Differences and associations between or among variables were considered significant when *P*-value is <0.05.

### Statistical analysis for gene interactions

Epistasis was evaluated by multifactor-dimensionality reduction analysis was performed using the MDR 3.0.2 software (Computational Genetics Laboratory, Institute for Quantitative Biomedical Sciences, Dartmouth, NH, USA)^3^ (Ritchie et al., 2003). The best model to predict the susceptibility to dementia was screened based on the minimum classification error in the training set, and for evaluating the predictive accuracy, 10-fold cross-validation was applied.

All the statistical results were significant at *p* < 0.05. For risk assessment, the genotype combination was identified as a high or low risk depending on the proportion of cases to controls.

Finally, a dendrogram and circle graph were used to display the selected model based on information theory (Moore et al., 2006). All interactions between SNPs show a percentage of the entropy risk. A positive percentage score was designated as synergistic interaction, while a score of 0 or below was designated redundant or antagonistic. The better model showed that paired percentage score is greater than the individual score (Hahn et al., 2003). All genetic models were adjusted for age, sex, BMI, glucose, cholesterol, and triglycerides.

## Results

### Subject characteristics

The frequency of women was higher in both groups; however, there was no significant difference. Participants with dementia were older as compared to the control group (76.9 ± 7.9 vs. 71.5 ± 7.8, *p* < 0.001).

There were significant differences between the dementia patients and controls regarding biochemical indicators. They showed higher glucose levels of 110 (95–148), total cholesterol of 247.0 ± 31.6, HDL-c of 48.0 ± 9.8, and triglycerides of 167 (132–240), all with *p* < 0.001 as shown in Table 2.

**TABLE 2:** Clinical and biochemical features of patients with dementia and control group.

Features	Control (*n* = 710)	Dementia (*n* = 221)	*P-value*
			
Gender, *n* (male%/female%)	270 (38.01)/440 (61.99)	76 (34.39)/145 (65.61)	*0.348*
Age (years)	71.5 ± 7.8	76.9 ± 7.9	<0.001
Education level [years (SD)]	6.9 ± 5.2	5.7 ± 5.5	0.005
BMI (kg/m^2^)	27.4 ± 5.0	26.4 ± 5.4	<0.001
Glucose (mg/dL)	98 (90–109)	110 (95–148)	<0.001
Total cholesterol (mg/dL)	202.6 ± 41.2	247.0 ± 31.6	<0.001
HDL-cholesterol (mg/dL)	52.8 ± 14.0	48.0 ± 9.8	<0.001
LDL-cholesterol (mg/dL)	117 (95–140)	121 (101–150.5)	0.002
Triglycerides (mg/dL)	146 (111–196)	167 (132–240)	<0.001

BMI, body mass index; SD, standard deviation; HDL, high-density lipoprotein; LDL, low-density lipoproteins.

### Association between dementia and the polymorphisms

The genotypic and allelic frequency distributions of each polymorphism are shown in Table 3. We found that IL-1a rs1800587 is associated with the susceptibility to developing dementia (OR = 1.55, 95%; CI = 1.20–2.00; *p* = 0.001). Similarly, IL-6 rs1800796 showed a risk effect on the susceptibility to dementia under the additive model (OR = 2.066, 95%; CI = 1.51–2.82; *p* = 0.001). In TNF-α, the strongest association was found using a recessive model with rs361525 (OR = 3.16, 95%; CI = 2.10–8.08; *p* < 0.001) and rs1800629 (OR = 4.39, 95%; CI = 2.10–9.02; *p* < 0.001). Finally, IFN-γ rs2069705 showed a protective effect on the susceptibility to dementia in subjects with the G allele vs. carriers of the A allele, under an overdominant inheritance model (OR = 0.59, 95%; CI = 0.43–0.81; *p* = 0.001). The models were independent of the Hardy–Weinberg equilibrium. Additionally, models were adjusted for the variables which were significantly different between groups and had been linked to dementia in previous studies.

**TABLE 3:** The genotypic and allelic frequency distributions of the genes included in the study.

Polymorphisms	Controls (*n* = 710)	Dementia (*n* = 221)	Model inheritance	OR (95% CI)^a^	*P-value*
**IL1-a **rs1800587****					
GG	486 (68.45)	127 (57.47)	Codominant	1.48 (1.07–2.05) 2.66 (1.36–5.18)	0.018 0.004
GA	201 (28.31)	78 (35.29)	Dominant	0.62 (0.45–0.84)	0.003
AA	23 (3.24)	16 (7.24)	Recessive	1.11 (0.83–1.48)	0.460
**G**	1173 (82.6)	332 (75.1)	Over-dominant	1.38 (1.00–1.90)	0.048
**A**	247 (17.4)	110 (24.9)	**Additive**	**1.55 (1.20–2.00)**	0.001*
**IL6 **rs1800796****					
GG	195 (27.46)	97 (43.89)	Codominant	0.55 (0.40–0.7) 0.32 (0.19–0.52)	0.001 <0.001
GC	353 (49.72)	98 (44.34)	**Dominant**	**2.06 (1.51–2.82)**	<0.001*
CC	162 (22.82)	26 (11.76)	Recessive	0.45 (0.28–0.70)	<0.001
**G**	743 (52.3)	292 (66.1)	Over-dominant	0.80 (0.59–1.09)	0.163
**C**	677 (47.7)	150 (33.9	Additive	0.56 (0.45–0.70)	<0.001
**TNF-a **rs361525****					
GG	642 (90.42)	164 (74.21)	Codominant	1.05 (0.60–1.85) 3.32 (2.17–8.03)	0.849 <0.001
GA	63 (8.87)	17 (7.69)	Dominant	0.30 (0.20–0.45)	<0.001
AA	5 (0.70)	40 (18.10)	**Recessive**	**3.16 (2.10–8.08)**	<0.001*
G	1347 (94.9)	345 (78.1)	Over-dominant	0.85 (0.48–1.49)	0.585
A	73 (5.1)	97 (21.9)	Additive	3.11 (2.34–4.13)	<0.001
**TNF-a **rs1800629****					
**GG**	634 (89.30)	154 (69.68)	Codominant 2	1.20 (0.71–2.01) 7.34 (16.7–13.5)	0.488 <0.001
**GA**	72 (10.14)	21 (9.50)	Dominant	0.27 (0.19–0.40)	<0001
**AA**	4 (0.56)	46 (20.81)	**Recessive**	**4.39 (2.10–9.02)**	<0.001*
**G**	1356 (95.5)	329 (74.4)	Over-dominant	0.93 (0.55–1.55)	0.782
**A**	64 (4.5)	113 (25.6)	Additive	3.43 (2.60–4.52)	<0.001
**IFNG **rs2069705****					
GG	224 (31.58)	86 (38.91)	Codominant 2	0.60 (0.42–0.85) 1.02 (0.68–1.53)	0.004 0.898
GA	349 (49.15)	81 (36.65)	Dominant	1.38 (1.01–1.89)	0.043
AA	137 (19.30)	54 (24.43)	Recessive	1.35 (0.94–1.93)	0.099
G	797 (56.1)	253 (57.2)	**Over-dominant**	**0.59 (0.43–0.81)**	0.001*
A	623 (43.9)	189 (42.8)	Additive	0.95 (0.77–1.81)	0.689

^a^Adjusted for age, sex, BMI, glucose, cholesterol, and triglycerides.

Genotype frequency is in (%).

*The significant associations according to the criteria of selection of the best inheritance model (AIC).

CI, confidence interval, IL, interleukin; TNF-a, tumor necrosis factor-alpha; IFNG, interferon-gamma.

Bold numbers indicate significant associations.

### Gene–gene interactions according to multifactor-dimensionality reduction analysis

The MDR identified the interaction between TNF-α rs1800629, TNF-α rs361525, IFNG rs2069705, IL1-a rs1800587, and IL6 rs1800796 as the best prediction model with an accuracy of 72.2%, a significance of *p* < 0.0001, and cross-validation of 10/10. The models with 2, 3, and 4 interactions showed a decrease in precision, with statistical significance. Only the five-locus model gave the highest sensitivity and specificity. Table 4 shows the results for each SNP.

**TABLE 4:** Analysis of interactions to measure dementia susceptibility.

Model	Accuracy (%)	Sensitivity (%)	Specificity (%)	Consistency	OR (95% CI)	*P-value*
TNF-a **rs1800629**	74.54	25.84	94.69	10/10	6.21 (3.73–10.35)	<0.0001
TNF-a **rs1800629** and TNF-a **rs361525**	73.23	35.75	88.74	7/10	4.38 (2.92–6.57)	<0.0001
TNF-a **rs1800629,** TNF-a **rs361525,** and IFNG **rs2069705**	73.42	45.60	84.94	10/10	4.72 (3.24–6.87)	<0.0001
TNF-a **rs1800629,** TNF-a **rs361525,** IFNG **rs2069705,** and IL1-a **rs1800587**	72.20	56.11	78.86	5/10	4.76 (3.34–6.80)	<0.0001
TNF-a **rs1800629,** TNF-a **rs361525,** IFNG **rs2069705,** IL1-a **rs1800587,** and IL6 **rs1800796**	72.26	68.48	73.82	10/10	6.12 (4.26–8.79)	<0.0001

Summary of multifactorial dimensionality reduction analysis (MDR) for dementia.

Bold numbers indicate significant associations.

**FIGURE 1:**
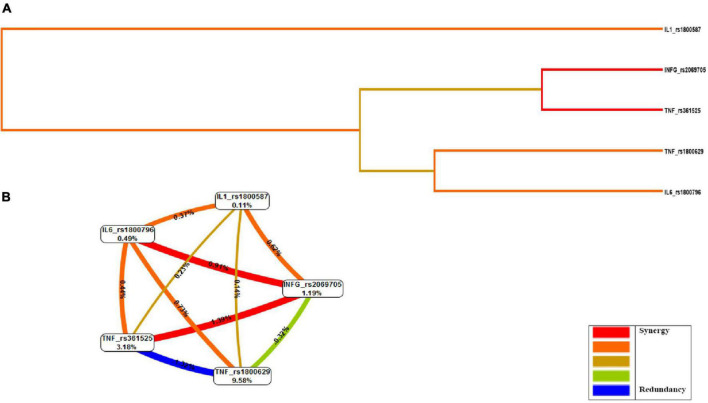
Evaluation of gene–gene interactions: multifactorial dimensionality reduction analysis (MDR). **(A)** Dendrogram, **(B)** Fruchterman-Rheingold graph. MDR combined attribute network was calculated. The interaction I (A; C) is represented as a percentage (%) between the five vertices. This graphical model explains the percent of the entropy in case-control removed by each factor (independent effect) and by each pair-wise combination of attributes (interaction effect). A positive percentage of entropy indicates synergistic interaction and negative values of entropy represent redundancy. The red color indicates a high degree of synergistic interaction, orange indicates a lesser degree, whereas gold represents the midpoint; blue represents the highest level of redundancy, followed by green.

We could see from the circle diagram information scores that the TNF-rs361525 percentage score (3.18%) was the most important among all gene variants in the study. IFN-γ-rs2069705 and TNF-rs361525 SNPs’ scores, shown in red for the interaction between the two, proved to be more significant than the individual contributions of each (Figure 1).

**FIGURE 2:**
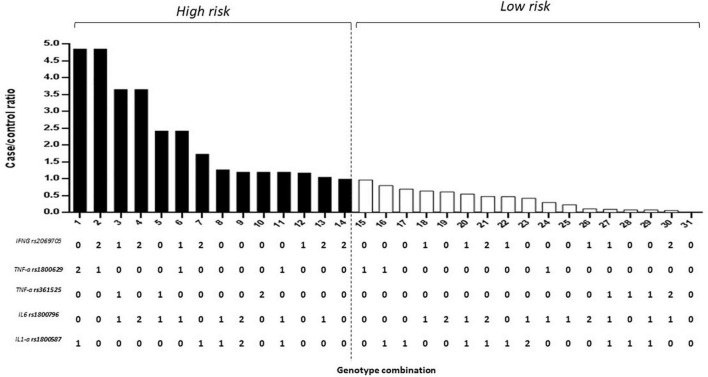
Five-locus gene–gene interaction between IL-1α (rs1800587), IL-6 (rs1800796), TNFα (rs361525 and rs1800629), and IFNγ (rs2069705) for dementia. The bar graph shows the validation of high and low-risk classifications. Genotype coding is as follows: 0, homozygous common allele; 1, heterozygous; and 2, homozygous variant allele.

Of the estimated genotypic pools, individuals with homozygous alleles for the IL-1α (rs1800587), IL-6 (rs1800796), TNFα (rs361525), and IFNγ (rs2069705) genes were identified in the high-risk group for dementia. The model was considered high risk when the ratio of the percentage of cases vs. controls was greater than 1.05. Fourteen genotype combinations resulted in a significantly high risk for dementia (Figure 2).

The combination that gave the highest risk (5-fold) was the haplotype heterozygote for the IL-6 (rs1800796), TNFα (rs361525), IFNγ (rs2069705), heterozygous IL-1α (rs1800587), and homozygous variant allele TNFα (rs1800629). Similarly, the combination of heterozygote for the IL-1α (rs1800587), IL-6 (rs1800796), and TNFα (rs361525) with heterozygous for TNFα (rs1800629) and the homozygous variant allele for IFNγ (rs2069705) with risks from 4.90.

When including in the analysis the MDR (gene-environment interaction) of the risk factors age, sex, BMI, glucose, cholesterol, and triglycerides, the best prediction model (*p* < 0.0001) maintained the same interaction model.

## Discussion

Dementia is a multifactorial disease; therefore, it is necessary to propose risk prediction models that consider both genetic and environmental aspects. The MDR analysis has already been used in association studies of other complex diseases such as diabetes, hypertension, and coronary heart disease, among others (Moore and Williams, 2002; Cho et al., 2004). The MDR allows us to detect and characterize patterns of epistasis in the genetic association (Cordell, 2009).

Previously, we reported the possible association of the IL10 (−1,082 and −819) polymorphisms in conferring dementia in a Mexica population (Vargas-Alarcon et al., 2016). In this study, the independent association between the genotypes (TNF-α rs1800629, TNF-α rs361525, IFNG rs2069705, IL1-a rs1800587, and IL6 rs1800796) was confirmed in the multilocus epistasis analysis, which may indicate that, at least in this population, the combination of these polymorphisms could constitute a genetic risk marker for the development of dementia. Previous studies have evaluated the association between some single-nucleotide polymorphisms (SNPs) in inflammatory cytokine genes and susceptibility to dementia, but the results are not conclusive. Some studies have found a significant association and others have not (Ravaglia et al., 2006; Di Bona et al., 2009; Swardfager et al., 2010; Hua et al., 2012; Toral-Rios et al., 2015; Sathyan et al., 2017). These inconsistencies can be attributed to the small effect of polymorphism on the disease, the small sample sizes, and the methodologies used.

From the pathophysiological point of view, the relationship between cytokines and dementia is established. The overexpressed IL-1 restrains the function of cholinergic systems (Li et al., 2000) and favors the formation of Aβ plaques and accumulation of neurofibrillary tangles (Sheng et al., 2001; Griffin and Mrak, 2002). This has been supported by gene association analysis. The relationship between the IL1A-889 C/T polymorphism, with dementia, has been tested in different ethnic groups from different regions, and the results vary (Combarros et al., 2002, 2003). Our study identifies the allele of IL1-α rs1800587 associated with the risk of dementia, which can facilitate the neuroinflammatory processes in the pathology.

Associations between dementia and IL-6 suggest that overexpressed IL-6 influences the cdk5/p53 pathway to induce the phosphorylation of tau, increasing the dementia risk (Quintanilla et al., 2004). Regarding the polymorphisms of the IL-6 gene, its protective role has been highlighted (Fishman et al., 1998; Darweesh et al., 2018). However, inconsistent results have also been reported (Fishman et al., 1998). Several meta-analyses have suggested that the CC genotype is likely a protective factor for dementia (van Oijen et al., 2006; Qi et al., 2012). Our finding supports a protective effect of the C allele in the IL-6 gene on dementia risk, which can favor the liberation of IL-6 protein release in the brain, playing an important role in the neuroinflammatory cascade and the pathology.

Increased TNF-α increases the Aβ and tau pathology and mediates the microglial phagocytosis of Aβ (Janelsins et al., 2008; Montgomery et al., 2011). Concerning genetic polymorphisms, some SNPs have been reported to influence the risk of dementia. A meta-analysis supported the association between the TNF-α rs361525 polymorphism and dementia risk involving different ethnic groups (Manoochehri et al., 2009; Ribizzi et al., 2010; Flex et al., 2014). Our study also identified the presence of the allele G in this polymorphism with a significantly increased risk of dementia. This genetic variant could modulate TNF-α expression and promote Aβ deposits which, in consequence, induce neuronal death (Di Bona et al., 2009).

The interferon (Abbas et al., 2002) activated by brain microglia exacerbates neurodegeneration, is neuroprotective, and helps clear neuronal debris and Aβ. Recently, some alleles in the first intron of the IFN-γ gene are associated with a higher level of cytokine production *in vitro* (Yamamoto et al., 2007). Our results showed a protective effect for dementia with the dominant over a model for the G allele, complementing existing information on the role of IFN-γ in neurodegeneration.

In Mexico, less has been explored about the relationship of the genes’ pro-inflammatory cytokines released from activated microglia (IL-1α, IL-6, TNFα, and IFNγ) with dementia. In our study, four of the polymorphisms studied showed an independent risk associated with dementia. However, in the results of the multilocus analysis, we could consider that the genes studied are not sufficient but necessary for the development of dementia, at least in this population. In this sense, MDR analysis can be useful as an analytical strategy to investigate the simultaneous effects of different loci and thus detect risk relationships with complex diseases, such as dementia.

**FIGURE 3:**
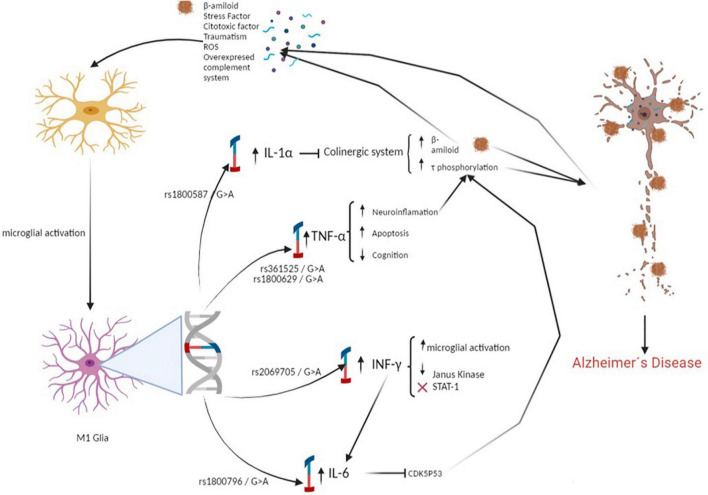
Polymorphisms associated with the development of dementia increases the proliferation of β-amyloid and promotes Tau hyperphosphorylation. Causing a pathological increase in the inflammatory response, which generates neuroinflammation and neuronal death.

Regarding the epistasis analysis, the MDR identified the interaction between the IL-6 (rs1800796), TNFα (rs361525), and IFNγ (rs2069705) polymorphisms as the best model. In contrast to the high- and low-risk genotypes obtained, we can point out that the polymorphisms studied can act synergistically: subjects carrying the genotypic combination will have a greater susceptibility to developing dementia, while those who do not present this combination could be protected (Figure 3).

A possible explanation for the combined effect of these SNPs could be in the relationship that the enzymes encoded by these genes have in the immunological pathways involved in protection against neurotoxicity, thus contributing to tau phosphorylation and accumulation of Aβ deposits, which, in consequence, induces neuronal death (Di Bona et al., 2009).

Another explanation could be that the combination likely affects the transcriptional efficiency of these cytokines by modifying their activity levels and promoting the circulation of inflammatory proteins in the body. This could lead to increased Aβ deposits and a decrease in the elimination of this protein in the brain.

The difference between our results and others previously reported for these polymorphisms can be explained by the ethnic characteristics of the populations. This could lay the groundwork for new therapeutic targets based on ethnic characteristics.

## Limitations

This study only analyzed the SNPs that had previously been reported. Thus, other SNPs can potentially show genetic interactions and cause dementia. Other studies to characterize the interactions with other SNPs are necessary.

Overall, our model was based on the integration of both main effects and epistasis to increase the predictive value of genetic risk. Finally, a more detailed analysis should be conducted in other populations to evaluate the effect of race on this relationship.

## Conclusion

The genetic contributions to the susceptibility to dementia were visualized thanks to computational techniques. We identified the strongest interactions between combinations of SNPs (IL-6 rs1800796, TNFα rs361525, and IFNγ rs2069705) associated with the risk of suffering from dementia. Furthermore, we identified and characterized patterns of epistasis in the genetic association between dementia and cytokines through the algorithm, including MDR. In combination with clinical factors, our findings can potentially help to better understand the pathophysiology of dementias and confirm the role cytokines seem to play in the predisposition and development of the disease.

## Data availability statement

The original contributions presented in this study are included in the article/supplementary material, further inquiries can be directed to the corresponding author.

## Ethics statement

The studies involving human participants were reviewed and approved by the National Commission of Scientific Research, as well as by the IMSS Ethics Commission. The patients/participants provided their written informed consent to participate in this study.

## Author contributions

TJ-C participated in the conceptualization, acquisition of funds, and wrote and edited the manuscript. NM-R participated in the methodology, analysis, and wrote the manuscript. GV-A participated in the methodology and analysis. EJ-C and OG-A participated in the methodology and supervision of the work. AV-M participated in the supervision and analysis. AR participated in the investigation and reviewed and edited the manuscript. All authors contributed to the article and approved the submitted version.

## Footnotes

1
http://www.ncbi.nlm.nih.gov/SNP

2
http://www.appliedbiosystems.com/

3
https://www.epistasis.org
